# Authentic Youth Engagement in Environmental Health Research and Advocacy

**DOI:** 10.3390/ijerph18042154

**Published:** 2021-02-23

**Authors:** Kathryn M. Cardarelli, Melinda Ickes, Luz Huntington-Moskos, Craig Wilmhoff, Angela Larck, Susan M. Pinney, Ellen J. Hahn

**Affiliations:** 1 College of Public Health, University of Kentucky, Lexington, KY 40504, USA; 2 College of Education, University of Kentucky, Lexington, KY 40504, USA; Melinda.ickes@uky.edu; 3 School of Nursing, University of Louisville, Louisville, KY 40202, USA; luz.huntingtonmoskos@louisville.edu; 4 Buckhorn High School, Buckhorn, KY 41721, USA; craig.wilmhoff@perry.kyschools.us; 5 College of Medicine, University of Cincinnati, Cincinnati, OH 45267, USA; larckan@ucmail.uc.edu (A.L.); pinneysm@ucmail.uc.edu (S.M.P.); 6 College of Nursing, University of Kentucky, Lexington, KY 40504, USA; ejhahn00@mail.uky.edu

**Keywords:** youth, environmental health, citizen science, empowerment, engagement, advocacy, community-based participatory research, science communication

## Abstract

Training in environmental health (EH) engages and inspires youth to tackle health promotion and policy change. Yet, there is little guidance on how to successfully nurture and sustain youth engagement. This paper compares four case studies of youth engagement to promote EH in rural and urban communities using the Youth Empowerment Solutions (YES!) framework. Of the case studies in rural (Central Appalachia) and urban (Cincinnati, Ohio) communities, two employ citizen science approaches using PhotoVoice and environmental sampling; one engages youth in a science communication camp; and one focuses on policy advocacy. We compare and contrast these case studies using the YES! Critical Components and Empowerment levels. The case studies were discussed at the 2020 Partnerships in Environmental Public Health Meeting, where participants identified challenges and possible solutions for promoting and maintaining authentic youth engagement in EH research and advocacy. Analysis of the case studies indicated that youth engagement activities focusing on the individual were more common than those targeting the organizational setting or the community. Youth demonstrate agency to impact EH issues in their communities by engaging in hands-on opportunities to practice citizen science and advocacy. Overcoming challenges to authentic young engagement is important to sustain this work.

## 1. Introduction

Engaging adolescents in the exploration of their health and the environment that surrounds them can result in enhanced knowledge and skill development, critical awareness, and opportunities for positive health behaviors and policy change. Yet, youth engagement is often seen as daunting, and it is not a one-size fits all approach. Developing effective and sustained youth engagement in environmental health can lead to great strides in health promotion, research and advocacy. Youth empowerment requires purposeful efforts to develop a sense of agency among adolescents; the sense that their efforts can make a difference [1]. It also requires practicing learned skills in real-life settings [2]. Adults who serve youth can foster this agency and skill development through authentic youth engagement, which can occur at individual, organizational and community levels. For the purposes of this paper, we define authentic youth engagement as providing meaningful opportunities to practice skills (e.g., leadership) in real-life settings and recognizing youth voices as valuable, with the goal of instilling a sense of confidence that their efforts can make a difference [3].

Youth engagement is the purposeful, collaborative work that occurs among groups working in partnership to address an issue that binds them [4]. The community engagement process can be viewed as a continuum where the partnership can move through the stages of outreach, consult, involve, collaborate and shared leadership. The beginning stage of outreach offers the opportunity to establish communication between groups and share information, while the final stage of shared leadership offers greater complexity of interaction and leads to decision making that is firmly in the hands of the community [4]. Partnerships can actively move along the continuum or they can exist in a stage that suits their stated goals. When implementing engagement in the research enterprise, we can further describe the approach through the use of community-engaged research and citizen science. Community-engaged research is an approach that begins with the research investigator who works to incorporate community input and perspective through engagement while citizen science originates in the community, often due to an issue or exposure of concern [5]. In the citizen science approach, community members (which can include youth) begin to engage in scientific questioning and seek out collaborative research partnerships [5,6,7]. Authentic youth engagement involves the structured implementation of the community engagement process with the goal of teaching youth about the shared leadership potential in a transparent manner so that youth can grow into full partners in the process.

Authentic youth engagement and subsequent empowerment result in three interrelated outcomes: (1) intrapersonal effects (e.g., beliefs of control, efficacy, and self-esteem); (2) interactional impacts (e.g., awareness of outside forces that shape one’s life and pursuits including support, resources, resource mobilization); and (3) behavioral effects, or the actions one undertakes to create change [8]. Adolescence, a developmental period of increased autonomy, identity formation and skill building, is an opportune time for positive health outcomes and future growth opportunities [1]. Youth empowerment efforts have been studied in many contexts [9,10,11], and youth empowerment approaches focused on environmental health encapsulate all three outcomes as adolescents gain: (1) confidence in their understanding of where environmental exposures exist and how they impact their health and the health of others; (2) understanding of the key community, industry and government stakeholders who drive the regulation of various chemical and other environmental exposures; and (3) opportunities to make change on an individual, organizational, and/or community level to improve health for all [12]. 

The literature describes engaging youth as research partners who are trained to collect environmental data related to air monitoring and personal chemical exposures [13,14,15]. However, little research exists that applies theory or evaluates methods on how best to nurture and sustain authentic youth engagement in environmental health research, communication and advocacy, which could ultimately lead to environmental health gains. Specifically, examining barriers and opportunities to overcome barriers using an established theoretical framework provides a unique opportunity to advance authentic youth engagement in environmental health. Building on Zimmerman’s Empowerment Theory [1], Youth Empowered Solutions (YES)) operationalized the theory to include implementation, effectiveness, and accountability of youth empowerment. YES! implements the theory of youth empowerment within the structure of the YES! Youth Empowerment Model (© 2021), an evidence-based, practical application of engaging youth in positive community change [1]. The YES! Youth Empowerment Model as a framework for examining youth engagement in environmental health is novel. The aims of this case study analysis were to: (1) compare the critical components of authentic youth engagement in environmental health promotion, science communication, advocacy and research using the youth empowerment model/framework; and (2) to highlight individual, organizational and community-level challenges and possible solutions to engaging youth in environmental health research and advocacy efforts.

## 2. Materials and Methods

### 2.1. Setting and Case Studies

The National Institute of Environmental Health Sciences (NIEHS) created its Partnerships for Environmental Public Health (PEPH) program in 2009 to foster community engaged research to improve environmental health. As part of the PEPH, a conference was held in February 2020 in Durham, North Carolina, to bring together NIEHS grantees and their partners to advance the science of community-engaged environmental health research. The meeting included multiple interactive educational workshops, including the one described here, which was organized by author E.J.H. The goals of the workshop were to (1) compare and contrast approaches to engage youth in environmental health promotion, science communication, advocacy, and research; (2) identify critical characteristics of effective youth-engaged approaches to reduce environmental health inequities in rural and urban environments; (3) identify ways to integrate citizen science and advocacy training into ongoing youth engagement efforts; and (4) identify resources and inspiration for youth engaged hands-on activities. 

During the PEPH workshop, at the direction of the workshop organizer (E.J.H.), participants (*n* = 60) engaged in small group discussions about the facilitating factors and barriers to engaging youth in environmental health research and advocacy efforts. The small group discussions were led by the case study leaders (authors K.M.C., C.W., A.L., M.I.), and multiple notetakers documented the discussions. Examples from the case studies were mentioned in the small group discussions, but the conversations also included broader examination of youth engagement challenges, benefits, and misconceptions. Workshop participants identified as teachers, youth-serving professionals from community agencies, scientists, and youth from diverse communities. Next, we held a report out session in which the small groups provided an overview of the major points discussed for all workshop attendees, and a broader discussion ensued. All notes and minutes from the workshop were reviewed by the authors, and themes were categorized according to the YES! Framework. The following case studies were shared for discussion in small groups during the workshop.

Mountain Air Youth Photovoice Project (PV). The objective of this PhotoVoice (PV) project was to engage youth in Appalachia to share their perspectives on environmental determinants of respiratory illness and involve them as catalysts for environmental health change through community engagement. PhotoVoice is a community-based participatory research (CBPR) approach designed to engage and empower individuals to learn the knowledge and skills needed to assist in transforming perspectives on pressing community concerns and inspire actions for positive public health change [16,17]. Ten participants (age 13–18) represented their perspectives about the environment and respiratory illness through photography and adjoining narratives over eight weeks. A brief thematic content analysis of the youth narratives that accompanied the photos revealed three primary themes of environmental determinants of respiratory illness. These themes included: compromises community members make regarding respiratory health in order to secure a livelihood; tension between cultural legacies and respiratory health; and consequences of geographic forces. This PV project demonstrated the value of deep understanding and analysis of environmental health concerns from the perspective of youth participation using PhotoVoice [18].

High School Students as Citizen Scientists (CS). The goal of the Citizen Science (CS) program was to equip high school students with the skills and knowledge needed to conduct research to address community concerns about environmental health in Appalachian Kentucky. The citizen science approach in the school setting was designed to train high school students with real-world experience in environmental science to analyze data and share it with peers and the community. Over a three-year period, over 100 high students from a rural Kentucky county were trained to monitor and report back air quality (ambient PM_2.5_) indoors and outside or trained in human subjects’ protection to consent homeowners to perform in-home testing for radon. The CS Program facilitated the following projects: (1) efficacy of engaging high school students as citizen scientists; (2) air quality on school buses using low-cost monitors [19]; and (3) in-home, short-term radon testing and report back in a rural Appalachian community. Students gained knowledge and skills to conduct research through participating in training on instrumentation use, data collection, report back, and ethical research practices. Students created a video and presented posters at local and regional public health and research conferences [20]. 

Medical Camp to Improve Science Communication (MC). The University of Cincinnati (UC) Center for Environmental Genetics (CEG) partners annually with Cincinnati Museum Center’s summer Medical Camp (MC) to increase knowledge about the human health implications of environmental exposures with 5th–8th grade urban youth. Medical Camp (MC) entailed two independent week-long sessions in the museum setting with a maximum enrollment of 24 participants per week. The CEG recruited and mentored graduate students from UC’s Department of Environmental and Public Health Sciences to create and lead the educational presentations with complementary hands-on learning activities to promote skill development with youth. The MC activities were bi-directional in the following ways: (1) emerging professionals developed real-world youth engagement communication skills and practice; (2) youth participants were empowered and inspired by the graduate students to analyze information and make decisions related to positive environmental health outcomes. In 2019, 74% of youth participants pinpointed hands-on activities as their favorite part of the Medical Camp. 

Youth Tobacco Advocacy Training Program (TA). The goal of the Youth Tobacco Advocacy Training Program (TA) was to train youth as advocates in order to promote tobacco control policies in Appalachian Kentucky. The project used the Youth Empowerment Theory as the guiding conceptual framework [3]. The TA Program was created during a community-engaged development phase (2017–2018) with 20 students from one Appalachian high school and revised during an implementation phase (2018–2019) with 80 students from three Appalachian high schools. An interdisciplinary team of community members, researchers, and college students helped develop and implement the program. Tailored and culturally relevant training provided information on tobacco use, consequences, industry tactics, evidence-based tobacco control, and advocacy skills. Students were also given the opportunity to participate in skill-building activities throughout the training and in monthly booster sessions, including community assessments and developing elevator speeches to talk with elected officials about policy change. Students reported improved self-efficacy, communication about policy advocacy, and greater support for tobacco policies [21]. 

### 2.2. Application of the Youth Empowerment Solutions (YES!) Youth Empowerment Model to Case Studies and Workshop Discussion

We used the (YES!) framework [22], which is an adaptation of the Youth Empowerment Theory [22], to compare the elements of authentic youth engagement in and across each of the four case studies as implemented. The YES! Framework is intended to build confidence in youth, to enhance critical thinking skills and to create positive change in their communities. Youth empowerment-based programs have focused on providing supportive contexts where youth build capacity, connect with local resources, and engage in community change activities [8,10,23]. YES! posits three critical components of effective youth engagement in meaningful efforts: skill development, critical awareness, and opportunities (see Table 1). The YES! framework acknowledges youth empowerment as a multi-level construct targeting the individual, the organization, and the community (Table 1). The YES! framework has been successfully applied with 92% of graduated YES! youth reporting they continued to not only be engaged in using their skills and critical awareness but also using them for civically minded purposes. Additionally, youth who participated in youth empowerment up to nine years ago reported a sustainable change in their behavior, including enhanced self-efficacy for public speaking, and taking advantage of leadership opportunities within their communities. 

The authors/case study leaders categorized the elements of their projects or programs according to the critical components and the empowerment levels. The author team each reviewed, made suggested revisions, and the case study leaders validated that the critical components and empowerment levels were accurately categorized for their project or program as it was implemented. In addition, relevant examples were included within each case study to further operationalize how the critical components and empowerment levels were applied across projects. Finally, we summarized PEPH workshop participant discussions of the barriers and possible solutions to authentic youth engagement in environmental health activities by empowerment level.

## 3. Results

### 3.1. Application of YES! To Case Studies

The four case studies of youth engagement in environmental health sciences revealed similarities and differences in the use of the YES! framework’s critical components and empowerment levels (see Table 2 for specific examples by case study). All four case studies used skill development and critical awareness activities at the individual level to impart deeper understanding and analysis to engage youth in environmental health. Opportunities, or actions to create community change through youth empowerment, were less commonly used. Three of the four case studies provided opportunities to take action for change at the individual level. However, only one case study (TA) engaged youth in taking action for change at the organizational level. The TA project engaged youth in local and state advocacy efforts through a letter writing campaign to elected officials and a media advocacy campaign to support tobacco treatment resources. 

Three of the four case studies used skill development and critical awareness activities to target the larger organizational system. Three of the four case studies used critical awareness activities at the community level, and two engaged youth in taking action for change via citizen participation. For example, the PV Project enhanced environmental health research skills of participating youth and built their capacity to communicate how their photographs represented risk factors for respiratory illness in a conference setting as well as at a community exhibit. The CS Program provided students the opportunity to create an informational video about their research, and present their research, experiences and views at a regional conference, which prompted local media stories. Only the TA case study used skill development at the community level by teaching youth how best to advocate for policy change and provide opportunities to do so with school stakeholders and elected officials. Regardless of the critical components, youth engagement activities focusing on the individual were more common than those targeting the organizational setting or the community. In addition, regardless of the empowerment level, youth engagement activities focusing on critical awareness were more common than those using skill development or opportunities for change.

### 3.2. Challenges and Possible Solutions to Authentic Youth Engagement

Discussion of these case studies by participants at the PEPH workshop focused on possible solutions to overcome some of these barriers. We organize the challenges and possible solutions below by empowerment level: individual, organizational, and community.

Individual level. Youth have varying literacy levels, learning styles, and energy levels, creating a challenge in engaging different age groups and those with individual learning needs. For example, in the *MC Project*, middle school aged children demonstrated high energy levels, which required thoughtful age-appropriate activities. Engaging high school youth can also be formidable given their level of involvement in other commitments and responsibilities. Students often have after school jobs and participate in clubs or sports. As a result, youth may not have time for additional projects. In addition, there may be a need for contact with parents, either for obtaining permission to participate or for implementing certain projects, creating an additional barrier to engaging youth. 

One possible solution to varying literacy levels and learning styles, the time barrier and need for parental contact might be to integrate the environmental health activity into the school curriculum and/or existing service learning or club activities. For example, the *CS Program* enlisted a local teacher (author C.W.) as its champion. The teacher’s daily access to students promoted communication; knowledge of school and student schedules; access to parents; and knowledge of age level appropriate language/learning tools. The champion’s mentoring relationship also reduced the challenge of keeping youth engaged; maintaining participation, even long-term if needed.

Another challenge is related to socioeconomic status (SES) and other disparities. For example, *MC* participants were limited by a hefty tuition fee for the week; digital learning tools, such as computers, tablets, and internet service, may not have been available to all students or organizations. Many projects may require transportation, and if youth participants do not have a car or other form of transportation, this reduces access to the activity. Possible solutions to these SES and other disparities include: (1) being flexible with students and leaders; (2) providing engaging, hands on activities rather than relying on digital tools; (3) being sensitive to the varying challenges of different cultural, ethnic, and racial backgrounds; and (4) listening, being respectful, and giving youth a voice in the project.

Organizational level. The culture, vision, and system (e.g., school or community organization) can also present a challenge in engaging youth in environmental health projects. Institutions must value youth contributions and youth voice. Schools and youth organizations likely have the greatest understanding of youth needs and abilities, but program or research staff members may not be as informed. On the other hand, school or youth organization staff may not be aware of the leadership value of allowing youth to participate in the process of designing and implementing all aspects of the project, which eventually informs their values and practices related to reducing environmental exposures and promoting environmental health [24]. For example, valuable training for school or organization staff may include setting structure and guidelines for participating youth, holding high expectations and reinforcing those expectations, and pushing youth toward their potential and providing feedback [24].

One solution for creating a supportive organizational culture, vision, and system to promote youth engagement is to implement age-appropriate programming. For example, in working with youth in the *MC Project*, it was important for university staff to incorporate a dynamic mix of didactic instruction and hands-on activities to account for the energy levels of middle school students. The classroom teacher can be a great asset in planning the pace of the session and suggesting methods for redirecting attention at times when the class may lose focus. In the *PV Project*, research staff found it effective to share their vision by giving examples of other youth research projects that prompted behavior changes in both students and their parents or caregivers. 

Another organizational challenge to engaging youth relates to logistics, such as difficulty obtaining access to the school building or changing student classroom schedules. In addition, some projects have eligibility requirements, such as the *CS Program* in which the parent had to own their home to take part in a home radon testing study [20]. In most cases, neither the teacher nor the student knew whether the home was owned or rented. Finally, navigating human subjects’ protection regulations may be a new concept for schools and other organizations serving youth. Many have never conducted human subjects research or worked with Institutional Review Board (IRB) oversight. Without a clear understanding of human subjects’ protections, which includes special protections for any person under the age of 18 years old, schools may not be transparent about the collection of identifiable data (e.g., drug and other controlled substance use) and not fully adherent to the principles of anonymity and confidentiality. 

Possible solutions to address these logistical organizational challenges are taking time to become familiar with the inner working of the larger system (e.g., school system or community organization) and ensuring that each level of the organization (including the administration) sees the benefit of the project or program. Knowing the organization can promote trust and expedite the project timeline and reduce unforeseeable issues. Teachers and other organizational leaders can often provide tips to communicating effectively with the upper administration of the school system or community organization. Project or program leaders may need to communicate the benefits in order to create administrative buy-in including direct monetary benefits, publicity, and increased opportunities for the students or the organization as a whole. 

Community level. Engaging youth in efforts to improve the community, respond to threats to quality of life, and provide for citizen participation at the local, state, and national levels can be a challenge especially if community outreach is beyond the scope of the organization’s mission. In addition, youth may not be recognized, valued, or supported as stakeholders in public health issues. Similarly, youth are likely not connected or linked to influential stakeholders or groups in the community. Furthermore, environmental health literacy at the community level may be relatively low [25], implying that the perceived value of youth engagement in environmental health projects or programs may be limited. 

Possible solutions to engaging youth and giving them a voice at the community level is to provide education on common environmental exposures, effective public health practices based in scientific principles, and advocacy skills, as in the *TA Project*. When engaging youth in any environmental health project designed with the community in mind, education about the issue and effective solutions must accompany any plan for data or information dissemination. Similarly, it is essential that all materials and methods be carefully prepared and revised with environmental health literacy in mind. Engaging youth in developing and testing literacy-appropriate materials for community report back, as in the *CS Program*, can be an effective method for improving community health. The community will not benefit from citizen science projects unless the findings are disseminated and reported back appropriately. Youth need the guidance of effective adult mentors who can connect them to influential stakeholders and organizations in the community. One of the best ways to provide youth a voice is to support them to develop confidence and the appropriate skill set to present findings to their school board or organizational leaders. These presentations provide opportunities to hear student voices and possibly influence change in their organization and community. In addition, youth need media advocacy training in order to communicate effectively with the community and participate in making change at the local, state, and national levels.

## 4. Discussion

We analyzed four youth engagement case studies using the Yes! Framework, and we discovered that skills development and critical awareness, primarily at the individual level, were the most common components of these youth engagement projects. Critical awareness activities involving analysis of information and resources were the most prevalent strategies used, followed by skills development. Opportunities, or actions for sustainable community change, were less commonly used approaches across the four case studies. We need to engage youth using strategies that provide opportunities to take action for sustainable community change and at the organizational and community levels in order to make the greatest impacts. Youth engagement projects that are most likely to make an impact promote empowerment at the individual, organization, and community levels and employ all three critical components: skill development, critical awareness, and opportunities [1]. 

Workshop participants identified several challenges to authentic youth engagement. At the individual level, participants identified that youth have varying levels of literacy, learning styles, energy levels, and socioeconomic status. If activities are costly and/or transportation is required, these may be barriers to youth engagement. In addition, some youth experience the competing demands of school, work, and/or extracurricular activities, limiting their time available to participate in environmental health projects. At the organizational level, participants shared that schools or other youth-serving organizations may not value youth leadership training nor may they have the adult expertise to promote it. Further, the program leaders may not have a good understanding of the organizational system and in the case of citizen science, the organization may not be familiar with regulatory issues (e.g., human subjects protection). At the community level, environmental health literacy may be low, impacting the perceived importance of youth engagement in these projects. Given that community outreach may not be consistent with the school or organization’s mission and youth may likely not be connected to key stakeholders, engaging youth in community level activities is a challenge. 

**Figure 1:**
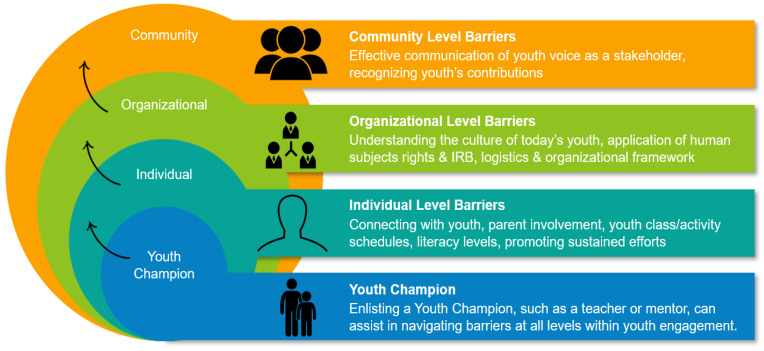
Overcoming challenges to authentic youth engagement.

Adults and organizations working with youth can mitigate many of these challenges by enlisting and training youth champions such as teachers or community organization leaders who work daily with youth (Figure 1). Teachers and youth leaders often have daily access to students for communication; a working knowledge of school and student schedules; ability to communicate with parents for granting permission for youth involvement; and knowledge of age-appropriate language/learning tools. Teachers and youth leaders develop longstanding mentoring relationships with students and can help them become engaged in citizen science or other environmental health projects. Other tactics for overcoming challenges to youth engagement in environmental health projects at the individual level are: (1) being flexible with students and teachers/leaders; (2) providing engaging, hands-on activities; (3) being sensitive to the varying challenges of different cultural and ethnic backgrounds; and (4) listening, being respectful, and giving youth a voice in the research. Allowing teachers/leaders and students to become actively engaged in research decisions and not just data gathering allows them to become vested in the research. 

In addition to individual-level solutions, our findings support that schools and other youth-serving organizations can promote authentic youth engagement by investing in age-appropriate youth programming that identifies, trains, and incentivizes adults who are willing to allow youth to take leadership and ownership of environmental health projects. Further, project leaders must be familiar with and earn the trust of the school or organizational system in order to engage youth in advocacy and research. This is the value of having a youth champion in that they can help project leaders navigate the system. In order for youth to effectively engage in policy change at the community level, they need a well-trained adult leader who can teach and model advocacy skills, provide opportunities for youth to present to policymakers and media reporters, and design report back materials based on the environmental health literacy level of the community. 

Promoting authentic youth engagement in environmental health by applying the strategies outlined in this paper is consistent with the NIEHS Strategic Plan [26], the European Environment and Health Process (EEHP), an international policy platform of World Health Organization European member states [27], and the United Nations [28]. NIEHS supports outreach, communication, and engagement with multiple stakeholder communities including schools and organizations serving youth. EEHP’s European Environment and Health Youth Coalition, created in 2014, promotes environmental health education and advocates for youth participation and leadership in environmental health The United Nations Secretary-General’s Envoy on Youth advocates for the meaningful participation of youth in advancing global sustainable development. The four case studies evaluated in this paper are good examples of community-academic partnerships that aim to engage youth in environmental health science projects important to both the community and universities. A new generation of environmental health leaders are needed to address current and future challenges, including climate change. Creating opportunities for authentic youth engagement in environmental health has potential for promoting environmental health literacy more broadly. 

## 5. Conclusions

As the case studies demonstrate, authentic youth engagement requires careful consideration of the multilevel barriers. Successful engagement necessitates getting to know the community, communicating with stakeholders (including youth and youth champions), and routinely reinforcing mutually beneficial, respectful relationships. Members of academia must recognize the time burdens and demands that the project could potentially have on the community members. Ultimately, authentic youth engagement in health research and advocacy will not only develop the next generation of scientists but also advance environmental health sciences. Being introduced to activities such as PhotoVoice, air quality testing, and report back, as well as communication and advocacy strategies, can impact self-efficacy to participate in environmental health initiatives. 

## Figures and Tables

**Table 1:** Youth Empowerment Solutions (YES!) Youth Empowerment Model: Critical Components, Empowerment Levels, and Definitions [22].

Critical Component	Definition
Skill Development	The process of enhancing youth’s skills and knowledge to enact community change
Critical Awareness	Proficiency of youth to identify, analyze and communicate information to enact community change
Opportunities	Circumstances available to youth to enact community change
**Empowerment Level**	
Individual	Youth ability to act with agency, based on skills, critical awareness and knowledge
Organizational	Demonstration of the implementation of a culture and vision that supports youth engagement
Community	Demonstration of the ability of a community to actively engage in activities to promote positive change

**Table 2:** Application of the Youth Empowerment Model across Four Case Studies: Critical Components by Empowerment Level.

Levels	Critical Components	Photo Voice Project	Citizen Science Program	Medical Camp	Tobacco Advocacy
Individual	Skill Development	Making impactful photographs, narrating the potential links between environmental factors and respiratory illness	Utilizing air quality monitors and indoor radon test kits to examine environmental contaminants	Learning how to read ingredient lists on personal care products, e.g., shampoo, and where to look to learn more about ingredient safety	Deconstructing tobacco advertisements
	Critical Awareness	Learning and discussing the potential contributions of environmental factors to the high rates of respiratory illness in their communities	Learning and discussing the consequences of air quality and radon on health	Discussing why scientists research chemical ingredients, risks on youth health, accompanied by an activity where participants analyzed a sample personal care product.	Discussing tobacco-related consequences tailored to their community; investigate existing tobacco control practices
	Opportunities	Presenting photographs at a community exhibit and presenting at conferences	Developingan internet video media message and reporting findings at regional scientific meetings		Developing effective media messages
Organizational	Skill Development		Learning and developing skills (e.g., data collection and interpretation) necessary to incorporate research into youth’s educational opportunities	Offering a training exercise for emerging professionals to learn to effectively translate science to youth.	Conducting SWOT analysis – focusing on opportunities
	Critical Awareness		Training on ethical use of human subjects in research and review of data from studies	Evaluating presentations; evaluations are shared with presenters and camp/university staff for continual program improvement.	Reviewing youth survey data (behaviors, perceptions)
	Opportunities				Creating youth-adult partnership agreement; school grassroots activity planning and implementation
Community	Skill Development				Engaging in policy advocacy scenariosand action planning (setting goals)
	Critical Awareness	Acknowledging the agency of youth and the importance of their voice for community matters	Acquiring permission from homeowners to collect indoor and inground radon samples		Conducting community assessments; photovoice
	Opportunities	Engaging youth in environmental health matters deemed important by the community	Reporting findings of radon study to homeowner; Reporting the harmful health effects of Radon to homeowners		Letter writing campaign to elected officials; media advocacy campaign for funding/treatment resources

## Data Availability

No new data were created or analyzed in this study. Data sharing is not applicable to this article.
